# Conditions of Childbirth in India

**Published:** 1921-03-26

**Authors:** 


### CONDITIONS OF CHILDBIRTH IN INDIA.

By A CORRESPONDENT.

[*The following article is from the pen of a British medical man who is attached to a mission hospital in India. The practices it reveals would be incredible were they not vouched for by the writer of the article. And this is the people whose chosen leaders and prophets are conducting a campaign for the rejection of everything Western!*—Editor, The Hospital.]

In the last annual report of the Sanitary Commissioner with the Government of India, the following rates of infant mortality per thousand are given : City of Bombay, 409.6; City of Calcutta, 239; Punjab, 248.6; United Provinces, 215.73. The latest vital statistics for England and Wales give the rate of infant mortality per thousand as 80.

Over 50 per cent. of the infant mortality of India is ascribed to uncleanly midwifery, and in the majority of cases the infant dies of tetanus. In an abnormally high percentage of cases the mother dies of tetanus or puerperal septicæmia.

The Practice of Child Marriage.

First in order of time, though not in importance, of the various causes contributing to this regrettable state of affairs is the practice of child marriage. Childbirth is naturally a much more serious affair for an immature mother, and in the Punjab "inquiries into a large number of cases show that where the marriage of young people is consummated at an early age, a fairly large percentage of wives die of phthisis or other diseases of the respiratory organs, or from some gynæcological complication, within ten years of the consummation of marriage."

More important are the conditions of childbirth itself. There are primarily two factors to be considered. First that nearly 95 per cent. of Indian women are illiterate. They are totally uneducated, not merely from a scholastic point of view, but they are ignorant of the most elementary rules of hygiene and cleanliness. Secluded behind their purdahs, Hygeia passes them by, while Kali, the goddess of birth and life, whom they adore, reaps a dreadful harvest with her dripping knife. The second factor is fundamental. Accouchement with all related to it is regarded as an unclean event. The expectant mother, for days before, will collect her filthiest clothing, for, as the native mind is economical, no clean clothing will be allowed to become defiled until the puerperium is over. The lying-in room is usually dark and dirty.

Bad Local Conditions.

No ventilation whatever is allowed, as fresh air is considered to cause septicæmia. Any cracks beneath doors or around windows are carefully stuffed with rag with an ingenuity worthy of a better cause. The room is often lighted from a tiny cotton wick floating in a primitive vessel of oil. There is usually a charcoal fire vitiating the air. The darkest figure in this tragedy is the native midwife or dai. These dais are usually of the scavenger or sweeper caste. They arrive for their task wearing unspeakably dirty clothing, wearing bracelets and rings. Their hands are begrimed with dirt, and their heads alive with vermin. Women of this type attend the vast majority of confinements in India. The system of caste forbids any women other than outcastes from engaging in this work. Hence the hopelessness of the prospect. Before commencing their attention to their patient, these women rub their hands upon a part of the floor made sacred by the application of cow dung. Hence the tetanus. The dais are not content, even in a normal case, to allow nature to take her course. "Knowing nothing of the dangers of dirt and sepsis, the dai never has the slightest hesitation" in using unclean hands in aiding delivery of the child. The uterus is often infected before the child is born. Various implements are used by the dais. One English trained nurse reports, "I have noticed a rusty nail, an unspeakably dirty and blunt knife, also a piece of kerosene oil tin which had just before been used to peel vegetables. The less fortunate infants are treated by the application of various substances of which earth is a common constituent, with the result that the incidence of tetanus in infants is alarmingly high." Another nurse reports, "the child is fed on either fresh or condensed milk, often on a rag leading from the milk vessel. Sometimes it is given native wine." There is also another practice too loathsome to mention. The performances of the dais when labour is abnormal are better imagined than described. A ruptured uterus is comparatively common in such cases.

The Remedy.

To remedy this state of things the Victoria Memorial Scholarships Fund was instituted by Lady Curzon in 1903. The funds were to be applied to the training of the hereditary dai caste as opposed to midwives from other outcaste classes. The work is full of difficulties. The dai obstinately adheres to her traditional lore, and is convinced that no one can teach her anything. She is helped by the conservatism of Indian women, who are averse to anything in the nature of change. The hereditary dai is practically the only form of assistance that many women can afford, added to which the dai makes herself useful in the house, while a woman of a higher class might not. In spite of these difficulties headway has been made. Centres of training have been organised, and the dais have been either bribed or compelled to attend. In many municipalities a qualified midwife is attached to the local dispensary or hospital, and the dais urged to call her in both to normal and abnormal cases. No fee is charged for her assistance. This method has not been successful owing to the jealousy of the untrained dai. The Committee of the Victoria Memorial Fund urge four essentials in the effort to cope with the difficulties: (1) Supervision of work; (2) education of the public in the importance of care and cleanliness at confinements; (3) provision of the essentials for cleanly work to mother and dais either free of charge or at a fair market rate; (4) instruction of dais by lectures and books. A training school for health and maternity supervisors has already been opened in Delhi.

